# Levels of Physical Activity during School Hours in Children and Adolescents: A Systematic Review

**DOI:** 10.3390/ijerph17134773

**Published:** 2020-07-02

**Authors:** Alberto Grao-Cruces, María J. Velázquez-Romero, Fernando Rodríguez-Rodríguez

**Affiliations:** 1 Department of Physical Education, GALENO Research Group, Faculty of Education Sciences, University of Cadiz, Puerto Real, 11519 Cadiz, Spain; alberto.grao@uca.es (A.G.-C.); mariajesusvelazquezromero@gmail.com (M.J.V.-R.); 2 Biomedical Research and Innovation Institute of Cadiz (INiBICA) Research Unit, Cadiz, 11510 Cadiz, Spain; 3 IRyS Research Group, School of Physical Education, Pontificia Universidad Católica de Valparaíso, Valparaíso, 2374631 Valparaiso, Chile

**Keywords:** primary education, secondary education, school schedule, recommendations, students

## Abstract

Background: This systematic review determines the levels of physical activity (PA) during school hours in children and adolescents. Methods: Studies carried out from January 1987 to December 2019 were retrieved from four databases (Web of Science, Pubmed, Scopus and SportDiscus). Results: Twenty-nine studies were included in this systematic review. Most of them used accelerometers and showed that male and female children accumulated a mean of between 14 and 68 min of moderate-to-vigorous PA (MVPA) during school hours (3–22% of this daily segment), and male and female adolescents accumulated a mean of between 13 and 28 min of MVPA during this daily segment (3–8% of the school hours). Less than a quarter of children and adolescents reached the recommended 30 min of MVPA during school hours, with notable differences between sexes. Conclusions: These results suggest that the levels of PA during school hours are not enough, and consequently, schools should develop strategies for helping children and adolescents reach the school PA recommendation.

## 1. Introduction

High levels of physical activity (PA) in children and adolescents are recognized as an indicator of subsequent physical, social and mental health [1]. Consequently, children and adolescents are recommended to engage in at least 60 min of moderate-to-vigorous PA (MVPA) per day [2]. However, there is some evidence that many children and adolescents worldwide do not meet this recommendation [3].

Although it depends on the country’s educational system and school’s resources, schools have a certain potential to help children and adolescents reach the daily PA recommendations because millions of children and adolescents spend a large proportion of their awake hours in school [4,5]. However, sedentary activities are still the most common within the school setting [6]. The American Heart Association indicated that children and adolescents should perform at least 30 min of MVPA during school hours [4]. A few studies have measured the compliance rate of this recommendation and have obtained mixed results [7,8,9]. This recommendation, in fact, sets a goal in an absolute value (minutes of MVPA), and school hours are not the same in all countries [9,10,11,12]

Traditionally, school leisure time (i.e., time periods of noncurricular activities in school, such as recess, breaks or lunchtime) and physical education lessons have been considered the most propitious periods to help students meet the aforementioned school-based PA recommendations [13,14,15]. For this reason, levels of PA during both periods have received considerable attention and have been the subject of prior systematic reviews [16,17]. However, in most countries, recess time is limited (approximately 30 min per day), and physical education is given relatively little time in the curriculum (approximately 2 h per week) [18]. Moreover, recent studies have shown that less than half of recess time and physical education lessons are spent in MVPA [9,16,17] and that there are more ways to accumulate MVPA during school hours (e.g., breaks, lunchtime or during academic lessons) that have been undervalued to date [19].

To the best of our knowledge, there is no systematic review that examines the quantity of PA or MVPA that children and adolescents accumulate during school hours without intervention. Likewise, it is important to examine the proportion of school hours spent in PA or MVPA, especially because school hours often differ between countries. The findings on both issues could clarify whether the recommendation of 30 min of MVPA during school hours is being met and if it is adapted appropriately to countries with different school schedules. Therefore, the aim of this systematic review was to determine the levels of PA during school hours in children and adolescents.

## 2. Materials and Methods

This study follows the PRISMA guidance [20]. A comprehensive search of four databases (Web of Science, PubMed, Scopus and SportDiscus) for items published from January 1987 to the end of 2019 was undertaken. In addition, reference lists were snowballed for reports citing identified studies. Table 1 shows the search terms and search threads that were used: (i) physical activity (physical activity, exercise, sport); (ii) school (school time, school schedule, school hours, primary school, secondary school); and (iii) students (students, children, schoolchildren, adolescents, teenagers, youth).

### 2.1. Selection Criteria

Papers retrieved during the searches were checked against the following inclusion criteria: (i) full-text original report published in a peer-reviewed journal; (ii) the study participants had no physical disability or health problems that might limit levels of PA; (iii) conducted in school on children and adolescents aged 6–18 years; (iv) showed device-measured PA levels during all school hours (not only during recess time or physical education lessons) and without intervention; (v) indicated the quantity of school hours in the educational system or, failing that, this information was provided by the corresponding author; and (vi) written in English or Spanish. There was no exclusion criterion in relation to ethnicity.

### 2.2. Data Extraction and Reliability

The search was conducted by three independent reviewers (A.G.-C., M.J.V.-R. and F.R.-R.). They read the titles and abstracts of all articles retrieved. A meeting was held to resolve disagreements about eligibility. Information about the author, title, aim, sample size, age, country, design, measurement, school hours, main results and conclusions was extracted from all studies. The results of studies that met the selection criteria were screened for retrieval.

### 2.3. Assessment of Quality and Level of Evidence

The quality of the selected studies was scored using a quality assessment list, on the basis of other standardized assessment lists. The list consisted of six items dealing with the sample, measurement, design and reporting of results. Items were rated as follows: 2 = reported in full; 1 = partially reported; 0 = not reported or unclear. Total quality scores for the studies were calculated by summing the scores for individual items (range: 0–12) and were used to categorize the level of evidence provided: (i) studies were defined as high quality if they had a total score of 9 or higher; (ii) a total score of 5 to 8 was defined as medium quality; and (iii) a score of less than 5 was defined as low quality (Table 2). Three reviewers (AGC, MJVR and FRR) evaluated the quality of the studies, separately. A consensus meeting was arranged to sort out possible differences between reviewers.

## 3. Results

### 3.1. General Findings

**Figure 1:**
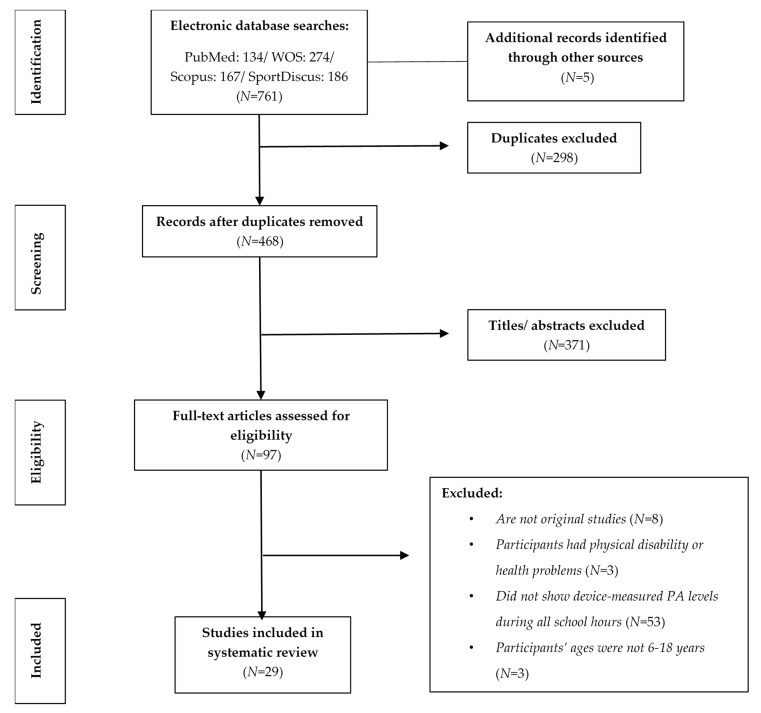
Flow of articles through the search process.

The flow of search results through the systematic review process is shown in Figure 1. The initial search retrieved 761 papers, and more than five identified in their reference lists, which was reduced to 468 by the removal of duplicates. The titles and abstracts of these 468 studies were screened, resulting in the exclusion of an additional 371 studies. In the last step, 68 papers were excluded because the population, age, language or design did not meet the inclusion criteria. Thus, 29 studies were included in the systematic review. Twenty-three (79%) of these were developed in primary schools, two (7%) studies were developed in secondary schools, and the rest (14%) included both primary and secondary schools. Twenty studies (69%) were cross-sectional, five (17%) studies involved interventions (only their baseline data were used, according to the selection criteria), three (10%) involved case studies, and one was a longitudinal study. Eleven (38%) studies were classified as being of high quality, seventeen (59%) were classified as medium quality, and one (3%) was classified as low quality (see Table 2).

This review covers data from 11,984 children and adolescents, of whom 9304 (77.6%) were primary school students and 2680 (22.4%) were secondary school students. The sample size of the studies varied from 21 [41] to 3040 participants [26]. The samples were from 20 different countries: six studies were conducted in the United Kingdom, three in the USA, two in Spain, two in Ireland, two in Switzerland, two in Hong Kong, one in France, one in Norway, one in Finland, one in Canada, one in Australia, one in New Zealand, one in Japan, one in Italy, one in Cyprus, one in Qatar, one both in France and Spain, one multicenter study in five European countries (Belgium, Greece, Hungary, Netherlands, and Switzerland). Table 3 and Table 4 show the main features of the selected studies with participants in primary school and secondary school, respectively.

### 3.2. Characteristics of the School Schedules

The number of school hours in the selected studies ranged in primary school from 240 min [22,26] to 480 min [11,24,34], with the most typical primary school time being approximately 360–390 min. In secondary school, the number of school hours ranged from 300 min [26] to 540 min [21] and in most cases was approximately 360 min. Twelve (41%) papers indicated the school leisure time. They showed that school recess ranged from 15 min [43] to 150 min per day [34], typically 30 min plus 60 additional minutes in schools with lunchtime. Twelve studies (41%) indicated the number of days per week with physical education lessons or if in the days measured there was physical education. In these studies, the weekly physical education time ranged from 44 min [10] to 240 min [9,33] and was typically 90–120 min distributed in one or two lessons.

### 3.3. Measures of Physical Activity

All selected studies included device-measured PA, of which 23 (79%) used accelerometers, four used pedometers (14%), one (3%) utilized both accelerometers and pedometers, and one (3%) used heart rate monitors with an integrated accelerometer. The studies that included accelerometer-based PA established different cut-off values to define the PA intensity categories. Most of these estimated the MVPA using cut-off values of ≥2000 counts per minute (cpm) or similar.

### 3.4. Absolute Physical Activity Levels during School Hours

Eighteen studies developed in primary schools showed the total PA as number of steps, cpm or minutes of PA. Children accumulated between 4358 steps and 7479 steps during school hours, without analyzing sex differences [11]. During that daily segment, boys accumulated from 3827 steps [30] to 8103 steps [42], and girls accumulated between 3420 steps [30] and 6963 steps [42]. Children’s cpm ranged from 129 cpm to 366 cpm on school days without and with physical education lessons, respectively [25]. According to sex, boys reached a mean of 145 cpm during school hours and girls reached a mean of 108 cpm [27]. Children spent between 104 min and 186 min in PA during school hours [11]. Boys accumulated from 86 min [33] to 178 min [43] in PA during school hours, whereas girls spent between 75 min [33] and 166 min of their school hours in PA [43].

Twenty-one studies developed in primary schools examined children’s MVPA during school hours. Children spent from 16 [8] to 61 min [11] of school hours in MVPA. The studies that analyzed their results separately by sex showed that boys accumulated between 20 min [43] and 68 min [42] of MVPA during school hours, while girls accumulated from 14 min [43] to 67 min [42] of MVPA during this daily segment.

Regarding secondary schools, three studies showed total PA data during school hours. Adolescents’ cpm during school hours ranged from 236 cpm [33] to 393 cpm [26]. Boys accumulated from 67 min [33] to 95 min [26] of PA during school hours, whereas girls spent between 54 min [33] and 76 min [26] of school hours in PA. The six studies that included secondary school students identified MVPA levels. During school hours, adolescents accumulated 23 min in Spain and 28 min in France [21] of MVPA. Boys spent between 16 min [23] and 26 min [26] of school hours in MVPA, whereas girls accumulated from 13 min [9,33] to 20 min [26] of MVPA during this daily segment.

### 3.5. Percentage of Physical Activity Levels during School Hours

Where the papers did not show the percentage of PA during school hours, we calculated this percentage wherever possible (e.g., % of school hours in MVPA = (minutes of MVPA/school minutes) × 100). In primary school, studies that did not analyze sex differences indicated that children spent from 4% [10] to 11% [41] of school hours in MVPA. The studies that analyzed their results separately by sex showed that boys spent between 5% [43] and 22% [42] of their school hours in MVPA, whereas this percentage for girls ranged from 3% [43] to 22% [42]. In secondary schools, adolescents spent between 4% [32] and 6% [21] of school hours in MVPA. Boys spent from 4% [23] to 8% [26] of their school hours in MVPA, whereas girls spent between 3% [9,23,33] and 6% [26] of their school time in MVPA.

### 3.6. Compliance with the School Physical Activity Recommendation

Four studies (14%) analyzed compliance with the school PA recommendation. In primary school, 7%–8% of European and American children reached the recommended 30 min of MVPA during school hours [8,36]. However, the studies that analyzed their results separately by sex showed higher compliance in boys than girls. Twenty-four percent of Spanish boys [9,33] and 8% of Spanish girls [9] met this recommendation. In secondary schools, 18% of Spanish boys and 2% of Spanish girls reached the 30 min of MVPA during school hours [9]. In both primary and secondary school, the percentage of compliance decreased with age [33].

## 4. Discussion

The aim of this systematic review was to determine the levels of PA during school hours in children and adolescents. A total of 29 studies conducted from January 1987 to the end of 2019 met the inclusion criteria and were heterogeneous with each other in their methods. Consequently, their results were also heterogeneous. They showed that male and female children accumulated a mean of between 14 and 68 min of MVPA during school hours, and male and female adolescents accumulated a mean of between 13 and 28 min of MVPA during this daily segment. Less than a quarter of children and adolescents reached the recommended 30 min of MVPA during school hours, with notable differences between sexes. These findings suggest that the current school settings might not generate a sufficient amount of MVPA in children and, especially, in adolescents. In spite of this, the results should be taken with caution because of the small number of studies (29), and only six studies included secondary school students. Moreover, the majority of the selected studies in the review were carried out in Europe, others in North America and in high-income countries from Oceania (Australia and New Zealand) and Asia (Japan, Hong Kong and Qatar). Most of them are developed and democratic countries, with a consolidated welfare state, where children and adolescents have access to education, in decent facilities and with teachers trained in universities. As a consequence, the results may not be generalizable to middle- and low-income countries.

Primary and secondary students’ PA levels were mixed between the selected studies. Only considering studies that employed an accelerometer—the most frequently used device in our systematic review—the average PA during school hours ranged from 75 min to 178 min in children and from 54 min to 95 min in adolescents. In these studies, children spent between 14 min and 68 min in MVPA, and adolescents spent between 13 min and 28 min. These levels of MVPA suggest that children spent between 3% and 22% of their school hours in MVPA and that adolescents spent between 3% and 8% of this daily segment in MVPA. Given that the school hours were not the same in all the selected studies, as we would expect, we attach particular importance to these percentage PA data. They confirm that PA levels are more varied in children than in adolescents. The most likely explanation is the higher number of studies developed in primary school among the selected studies, particularly because the methods of these studies are mixed (e.g., the study in which children reached the higher levels of PA [42] estimated the MVPA using a cut-off value of 1500 cpm, an amount significantly less than most of the studies).

Likewise, the data clearly show higher levels of PA during school hours in children than in adolescents. In secondary schools, it is most common to sit during academic activities [46], whereas in primary school, the environment is more propitious to active learning. Primary school students move more during non-curricular breaks [9,33] because there are more sport opportunities and greater encouragement from teachers [47].

However, these PA differences between educational stages are not only attributable to the schools, it is known that PA levels are influenced by a large amount of biological (e.g., age), behavioral (e.g., substance use), psychosocial (e.g., social support) and environmental factors (e.g., sport facilities) [48,49,50]. It is well documented that increasing age is a biological factor that is negatively associated with PA during childhood and adolescence [51,52], even without changing the educational stage [53]. It has been suggested that different social or behavioral factors may contribute to this decrease of PA with age [49], but it is not clear yet what set of reasons explains this change [49,54].

Our results also showed that boys were more active during school hours in both primary and secondary schools. Part of the reason for this is that boys accumulated more minutes of MVPA during school recess than girls [9,33], who spent more time performing sedentary activities [46]. This is evidence that transcends school hours and is due to socio-ecological factors at the individual, family, school, and environmental levels. Some individual attributes associated with PA are less favorable in girls than boys (lower cardiorespiratory fitness, lower eye–hand coordination, higher percent body fat and lower perceived competence in physical education). This is possibly due to the persistence of gender stereotypes, families are less supportive of girls’ PA than boys’ PA. Likewise, the opportunities for PA offered by school and community are more attractive to the boys than the girls [55].

Both age and sex differences were also observable in the scarce studies that analyzed compliance with the school’s PA recommendation. Less than a quarter of children and adolescents reach 30 min of MVPA during school hours, and this result is especially worrisome in adolescents and girls. However, many selected studies developed in primary schools (that did not show data on compliance with this recommendation) showed a mean MVPA during school hours higher than 30 min [24,26,28,37,41,42,44,45]. This might indicate that the situation in primary school is not so worrisome, but this is not the case for studies developed in secondary schools. In any case, this systematic review showed significant differences between the school schedules of the selected studies. Consequently, we think that a recommendation of PA within a school expressed in minutes is not the most advisable, because this goal would be easier to reach in schools with longer schedules. We propose a recommendation as a percentage of school hours, as is the case for the specific PA recommendation during recess [56,57] and PE lessons [58]. We understand that the American Heart Association established 30 min of MVPA for the American educational system, whose typical duration is 6 h per weekday [59]. These minutes suggest approximately 8% of school hours (not necessarily including a physical education session), which could be a realistic goal considering the results of this review.

There are different evidence-based strategies that schools can develop to help children and adolescents reach this goal of PA during school hours [9]. The results from the selected studies suggest that increasing the weekly hours of physical education and school recess would be useful for this purpose because children and adolescents are more active during these times than in other academic lessons [29,31,33]. Likewise, outdoor learning seems to be better for accumulating MVPA minutes than traditional indoor learning [41]. Moreover, other strategies could increase students’ PA during academic lessons (e.g., MVPA in the classroom while maintaining academic time [60,61]), school recess (providing access to outdoor school facilities and providing sports equipment [62,63,64]) and physical education lessons (reducing transition and management time during PEC [65]), and they are more viable.

This systematic review shows the heterogeneity in the methodology of the selected studies as its main weakness. Even between studies with the same design and devices to assess PA, there were differences in their data collection and processing criteria, which may bias the results. Some studies do not detail the number and duration of recess and physical education lessons, which may also influence the results. These issues make it difficult to compare the selected studies. Moreover, the review has some limitations linked to the device used to assess PA in the studies (mostly accelerometers), such as the lack of sensitivity to capture upper body movements, water-based activities, cycling or other complex movements. Another limitation was that the used quality assessment list does not have validation references, although it is conducted on the basis of other standardized assessment lists. Despite the above, this is the first systematic review to examine the quantity of PA that children and adolescents accumulate during school hours. Moreover, the possible bias due to differences between school schedules was minimized by providing percentages of PA in relation to school hours. Another strength is that the scope of this review was limited to studies that included device-measured PA and shows PA data during school from both primary and secondary schools. Future studies that focus on device-measured PA during school hours should use strategies that may help to ensure comparability between studies and maximize the potential for data harmonization [66,67]. Multilevel studies that examine school effects on children and adolescents’ PA during school hours [68] are also recommended to explain the differences observed between schools.

## 5. Conclusions

This review identified 29 studies of PA during school hours. Their methods and results were heterogeneous and indicated that children spent a mean of between 14 min to 68 min of MVPA during school hours, which represents between 3% and 22% of this daily segment, whereas adolescents accumulated a mean of between 13 min to 28 min of MVPA, a 3%–8% of the school hours. A very low percentage of children and adolescents reached the recommended PA levels for school hours, and consequently, schools should develop strategies to increase the levels of PA during school hours.

## Figures and Tables

**Table 1:** Search strategy in databases.

Database	Search Strategy	Limits	Filters
Web of Science	TITLE: (physical activity or exercise or sport) AND TITLE: (school time or school schedule or school hours or primary school or secondary school) AND TITLE: (students or children or schoolchildren or adolescents or teenagers or youth)	SCI-EXPANDED, SSCI Title Article English/Spanish	274 items filtered
PubMed	(((physical activity[Title] OR exercise[Title] OR sport[Title])) AND (school time[Title] OR school schedule[Title] OR school hours[Title] OR primary school[Title] OR secondary school[Title])) AND (students[Title] OR children[Title] OR schoolchildren[Title] OR adolescents[Title] OR teenagers[Title] OR youth[Title])	Title English/Spanish Humans	134 items filtered
Scopus	TITLE (physical AND activity OR exercise OR sport) AND TITLE (school AND time OR school AND schedule OR school AND hours OR primary AND school OR secondary AND school) AND TITLE (students OR children OR schoolchildren OR adolescents OR teenagers OR youth)	Title English/Spanish	167 items filtered
SportDiscus	TI (physical activity or exercise or sport) AND TI (school time or school schedule or school hours or primary school or secondary school) AND TI (students or children or schoolchildren or adolescents or teenagers or youth)	Title Article English/Spanish	186 items filtered

**Table 2:** List of included studies with quality scores.

Author	A	B	C	D	E	F	Total Score	Quality Level
Aibar et al. [21]	2	0	2	2	2	0	8	MQ
Bürgi et al. [22]	1	0	2	2	2	0	7	MQ
Bürgi et al. [23]	1	1	2	2	2	0	9	HQ
Cheung [10]	1	1	2	2	2	2	10	HQ
Clark et al. [24]	1	0	2	2	2	0	7	MQ
Dale et al. [25]	1	0	0	2	2	2	7	MQ
Dalene et al. [26]	2	2	2	1	2	0	9	HQ
Eyre et al. [27]	1	0	0	2	2	1	6	MQ
Fairclough et al. [28]	1	1	2	2	2	1	9	HQ
Fairclough et al. [29]	1	2	2	2	2	0	9	HQ
Galloway et al. [30]	1	2	1	2	2	1	7	MQ
Gao et al. [31]	1	0	0	2	2	1	6	MQ
Gidlow et al. [32]	1	2	2	2	2	0	9	HQ
Grao-Cruces, Segura-Jiménez et al. [33]	2	0	2	2	2	1	9	HQ
Grao-Cruces, Sánchez-Oliva et al. [9]	2	0	2	2	2	1	9	HQ
Guinhouya et al. [34]	1	0	2	2	1	2	8	MQ
Hardman et al. [35]	1	0	0	2	1	0	4	LQ
Hubbard et al. [36]	2	0	2	2	2	0	8	MQ
Kidokoro et al. [37]	0	0	1	2	2	0	5	MQ
Loucaides [38]	1	0	0	2	2	0	5	MQ
Martin and Murtagh [39]	1	2	2	2	2	0	9	HQ
Murtagh et al. [40]	1	0	0	2	2	0	5	MQ
Pau et al. [11]	1	0	2	2	2	0	7	MQ
Romar et al. [41]	0	0	2	1	2	2	7	MQ
Rush et al. [42]	0	0	2	2	1	0	5	MQ
Taylor et al. [43]	1	0	2	2	2	2	9	HQ
van Stralen et al. [8]	2	2	2	2	2	0	10	HQ
Watson et al. [44]	2	0	2	2	2	0	8	MQ
Zimmo et al. [45]	1	0	1	2	2	1	7	MQ

HQ: High quality; MQ: Medium quality; LQ: Low quality; A: Sample size (0: fewer than 50 participants; 1: 50 to 300 participants; 2: More than 300 participants); B: Is the sample statistically representative of a population? (0: no; 1: yes, but the sampling followed is not detailed; 2: yes, and the sampling followed is detailed); C: Are physical activity minutes differentiated by intensities? (0: minutes of physical activity are not shown; 1: only minutes of total physical activity are shown; 2: yes); D: Is the paper’s journal included on the JCR? (0: no; 1: no, but it is included on SJR; 2: yes); E: How many days wearing the device to measure physical activity? (0: 1 day; 1: 2–3 days; 2: more than 3 days); F: Does it indicate if there were physical education lessons on measurement days? (0: not reported; 1: the number of weekly sessions and their duration is reported; 2: it is specified for each day measured whether or not there was physical education).

**Table 3:** Characteristics of the analyzed studies with participants of primary school.

First Author	Study Design/Measurement Year/Country	Sample/Age	PA Measuring	Mean Data about School and PA	Results
Bürgi et al. [22]	Cross-sectional/2014/Zurich (Switzerland).	*N* = 83 (48.2% girls). *Average age (years):* 8.5 (7–9).	A. Actigraph GT3X; GPS receiver (BT-Q1000XT). From waking time to bedtime (7 consecutive days). Evenson et al. (2008), 101 ≤ LPA ≤ 2295 cpm; 2296 ≤ MVPA ≤ 4011 cpm; VPA ≥ 4012	School schedule: 4 h No reported PE sessions.	Average MVPA: 121.5 min/week.
Cheung, [10]	Cross-sectional/2015-16/Hong Kong.	*N* = 242 (45.9% girls). *Average age (years):* 8.7 (6–13).	A. Actigraph GT3X. All-day use. Remove only for bathing or swimming (7 consecutive days). Evenson et al. (2008), 101 ≤ LPA ≥ 2295 cpm; 2296 ≤ MVPA ≤4011 cpm; VPA ≥ 4012.	School schedule: 7 h 20 min. PE session: 35–60 min (44 min on average; 5%–8% of total school hours).	Average LPA and MVPA: 100.2 min and 18 min respectively. PE performance: 28.8 min of MVPA/day.
Clark et al. [24]	Case study/2010-13/Southwest Ontario (Canada).	*N* = 163. *Average age (years):* 10–12.	A. Actical^TM^. From waking time to bedtime (8 consecutive days). Puyau et al. (2004), MVPA > 1500 cpm.	School schedule: 8 h. A traditional day: (2 recesses × 15 min + 60 min lunchtime). A balanced day: (2 recesses × 40 min). No reported PE sessions.	Traditional and balanced average MVPA’s day: Boys: 44.9 min/d. Girls: 31.9 min/d. Girls’ PA< boys.
Dale et al., [25]	Cross-sectional/2009/Arizona (USA).	*N* = 78 (51.3% girls). *Average age (years):* 9.3 (7–15).	A. WAM Model 7164. From waking time to bedtime (4 school days). Trost, et al. (1998).	School schedule: 6 h. 2 days with no PE and no recess. 2 days with PE and 2 recess × 20 min.	Students spent on active and restricted days 366 cpm and 129 cpm, respectively.
Dalene et al. [26]	Cross-sectional/2011/Norway.	*N* = 2256 (51% girls). *Average age (years):* Group 6 = 6.6; Group 9 = 9.6.	A. Actigraph CT1M and GT3X. From waking time to bedtime (7 consecutive days). Andersen et al. (2006), 100 ≤ LPA ≤ 1999 cpm; MVPA ≥ 2000 cpm.	School schedule: 4 h. No reported PE sessions.	On average: 6 years old group: PA: 765 cpm. Boys’ LPA and MVPA: 88 min and 37 min. Girls’ LPA and MVPA: 85 min and 31 min. 9 years old group: PA 845 cpm. Boys’ LPA and MVPA: 76 min and 34 min. Girls’ LPA and MVPA: 71 min and 26 min. Girls’ PA< boys.
Eyre et al. [27]	Cross-sectional/2010/Coventry (UK).	*N* = 161 (96 Europeans + 65 Asians) (60.9% girls). *Average age (years):* 9 (8–9).	Monitor de FC Actiheart, Camntech, UK. All-day use (7 consecutive days). MET: 1.5 ≤ LPA < 3; 3 ≤ AFM < 6; VPA ≥ 6.	School schedule: 6 h 15 min. A recess × 15 min + 60 min lunchtime. PE session: 60 min 1 d/week for 60 min + 1 swimming day not supervised.	On average: European group: PA: 124 cpm. Asian group: PA: 120 cpm. Boys: 145 cpm. Girls: 108 cpm. Girls’ PA< boys.
Fairclough et al., [28]	Cross-sectional/2005/England (UK).	*N =* 58 (46.6% girls). *Average age (years):* 7–11 (8.6 boys and 8.4 girls).	A. Actigraph GT1M. During 5 consecutive days. Nilson et al. (2002), 1956 ≤ MVPA ≤ 5759 cpm; VPA ≥ 5760 cpm.	School schedule: 6 h. No reported PE sessions.	On average: Boys’ MVPA: 32.8 min/d. Girls‘ MVPA: 25.4 min/d.
Fairclough et al. [29]	Cross-sectional/2009/Wigan (UK).	*N* = 223 (98 low activity+ 125 high activity) (40.8% girls + 67.2% girls, respectively). *Average age (years):* 10.6 low activity; 10.7 high activity (10–11).	A. Actigraph GT1M. From waking time to bedtime (7 consecutive days). 2000 ≤ AFM ≤ 3999 cpm; VPA ≥ 4000 cpm.	School schedule: 6 h 30 min. PE session: 2 d/week (in class time, without any differentiation).	High activity group’s MVPA: 28.4 min/d. Low activity group’s MVPA: 23.3 min/d.
Galloway et al. [30]	Cross-sectional/2018/Mississippi (USA).	*N* = 241. *Average age (years): (9–10).*	A. Actigraph GT3X. From school to bedtime (5 consecutive days). Evenson et al. (2008), 101 ≤ LPA ≤ 2295 cpm; 2296 ≤ MVPA ≤ 4011 cpm; VPA ≥ 4012.	School schedule: 6 h. 29.4 min-recess/d PE session: 76.4 min/week and 17.4 min/d.	On average: Students’ MVPA: 19.8 min/d. Boys: 22.8 min/d. Girls: 17.1 min/d. Walking distance during school hours: Boys: 3827 steps. Girls: 3420 steps.
Gao et al. [31]	Cross-sectional/2014/Hong Kong.	*N* = 68 (58.8% girls). *Average age (years):* 10.4 (10–11).	SW 700 YAMAX Pedometer. From waking time to bedtime (4 consecutive days).	School schedule: 7 h. 3 × 20 min-recess/d + 60 min- lunchtime. PE session: 2 d/week (35 min).	Sample’s walking distance during school hours: 5110 steps. Boys: 5734 steps. Girls: 4672 steps. Girls’ PA< boys.
Gidlow et al. [32]	Cross-sectional/2006-07/England (UK).	*N* = 233 (49.8% girls). *Average age (years):* Primary 8.5 (5.4–11.7).	A. Actigraph GT1M. From waking time to bedtime (7 consecutive days). Trost et al. (2002) and Puyau et al. (2002), MVPA ≥ 3200 cpm.	School schedule: 6 h. No reported PE sessions.	Average students’ school hours PA and MVPA: 510.7 cpm and 24 min/d.
Grao-Cruces, et al. [33]	Longitudinal/2011-12/2013-14/Cadiz (Spain).	*N* = 814 (48.1% girls) **First measurement.** *Average age (years):* 8.1 (boys) and 8.2 (girls). **Second measurement.** *Average age (years):* 10.1 (boys) and 10.3 (girls).	A. Actigraph CT1M, GT3X and GT3X+. From waking time to bedtime (7 consecutive days). 100 < LPA < 2000 cpm; MVPA > 2000 cpm; VPA > 4000 cpm.	School schedule: 5 h. 30 min-recess. PE session: 2 d/week (60 min).	*First measurement:* boys’ PA: 483.2 cpm from which 76 min/d of LPA and 24.2 min/d of MVPA. Girls’ PA: 392.6 cpm from which 71.2 min/d of LPA and 18.4 min/d of MVPA. *Second measurement*: boys’ PA: 440.1 cpm from which 63.6 min/d of LPA and 22.5 min/d in MVPA. Girls’ PA: 341.7 cpm from which 58.6 min/d of LPA and 16.7 min/d in MVPA.
Grao-Cruces, et al. [9]	Cross-sectional/2011-12/Cadiz (Spain).	*N* = 926 (48.2% girls) *Average age (years):* 8.1.	A. Actigraph GT1M, GT3X and GT3X+. From waking time to bedtime (7 consecutive days). 100 < LPA < 2000 cpm; 2001 < AFM > 4000 cpm; VPA > 4000 cpm.	School schedule: 5 h. 30 min-recess. PE session: 2 d/week (45–120 min) or 1 d/week (90–150 min).	On average boys’ PA: 100 min/d and girls PA: 89.2 min/d. Boys’ MVPA: 24.1 min/d. Girls’ MVPA: 18.3 min/d. Girls’ PA < boys.
Guinhouya et al. [34]	Cuasi-experimental/2005-06/Lille (France).	*N* = 93 (40% girls). *Average age (years):* Children 10; Girls 10.1 (8–11).	A. Actigraph Model 7164. From waking time to bedtime (≥ 2 school selected days). MVPA ≥ 3200 cpm.	School schedule: 8 h 2 recesses × 15 min + 120 min lunchtime. PE sessions not included in the measurement.	On average, all participants at baseline: Boys’ MVPA: 29.9 min/d. Girls’ MVPA: 17.7 min/d.
Hardman et al. [35]	Cross-sectional/2003/Wales (UK).	*N* = 95 (54.1% girls). *Average age (years):* Boys 9.8; Girls 10 (7–11).	YAMAX SW-200 Pedometer. During ≥3 weekdays and 2 weekend days.	School schedule: 6 h 30 min. No reported PE sessions.	Average walking distance during school hours: Boys: 7312 steps. Girls: 5782 steps. Girls’ PA< boys.
Hubbard et al. [36]	Cross-sectional/2013-14/New England (USA).	*N* = 453 (60.5% girls). *Average age (years):* 9.1 (8–11).	A. Actigraph GT3X and GT3X. From waking time to bedtime (7 consecutive days). Evenson et al. (2008), 51 ≤ LPA ≤ 1148 cpm; 1149 ≤ AFM ≤ 2005 cpm; VPA ≥ 2006 cpm.	School schedule: 6 h 25 min. No reported PE sessions.	Average students’ PA: 161.5 cpm from which 143.8 min/d in LPA and 18.1 min/d in MVPA. Boys: 22.0 min/d. Girls: 14.2 min/d. Girls’ PA < boys.
Kidokoro et al. [37]	Cuasi-Experimental/2018/Japan.	*N* = 38 (42.1% girls). *Average age (years):* 11.3 (11–12).	A. ActiGraph wGT3X-BT. From waking time to bedtime (5 school days). Evenson et al. (2008), 101 ≤ LPA ≥ 2295 cpm; MVPA ≥ 2296 cpm.	School schedule: 7 h 40 min. No reported PE sessions.	At baseline: Average control class students’ MVPA during school hours: 40.5 min/d. Average intervention class students’ MVPA during school hours: 46.9 min/d.
Loucaides [38]	Cross-sectional/2016/Limassol (Cyprus).	*N* = 64 (54.7% girls). *Average age (years):* 11.5.	Yamax DW-200 Pedometer. For 6 winter days and 6 spring days.	School schedule: 5 h 20 min: 20 min-recess + 2 × 10 min-recess No reported PE sessions.	Average student’s walking distance: Winter: 5930 steps (Boys: 6795, Girls: 5223 steps). Spring: 6417 steps (Boys: 7068, Girls: 5884 steps).
Martin and Murtagh [39]	Experimental (Cluster randomised controlled trial)/2014/Limerick (Ireland).	*N* = 186. *Average age (years):* 8.9 (8–12).	A. Actigraph GT3X and GT3X+. During school hours (5 consecutive days). Evenson et al. (2008), 101 ≤ LPA ≥ 2295 cpm; MVPA ≥ 2296 cpm; VPA ≥ 4012 cpm.	School schedule: 5 h 40 min. No reported PE sessions.	At baseline: Control group LPA: 96.3 min/d, MVPA: 21.5 min/d. Intervention group LPA: 92.1 min/d, MVPA: 19.2 min/d.
Murtagh et al. [40]	Experimental/2010-11/Ireland.	*N* = 90 (45.6% girls) *Average age (years):* 9.3.	Yamax Digiwalker SW-200 pedometer. During all day (5 school days).	School schedule: 5 h30 min with a recess and a lunchtime. No reported PE sessions.	Average waking distance at baseline: Control group 5469 steps. Intervention group: 5351 steps.
Pau et al. [11]	Case study/2015-16/Cagliari (Sardinia, Italy).	*N* = 169 (55% girls). *Average age (years):* 8.6.	A. Actigraph GT3X. Durante all day (7 consecutive days). Crouter et al. (2015), 101 ≤ LPA ≤ 609 cpm; 610 ≤ AFM ≤ 1809 cpm; VPA > 1809 cpm.	Regular school schedule: 5 h with 15 min-recess. Full Schedule: 8 h with 15 min-recess + 60 min-lunchtime + 60 min-additional recess. No reported PE sessions.	On average, Regular Schedule students’ PA and MVPA: 104 min/d and 11% of school time (33 min/d). Full Schedule student’s PA and MVPA: 186.3 min/d and 9.5% of school time (60.9 min/d). Average walking distance: Regular Schedule student’s: 4358 steps. Full Schedule student’s: 7479 steps.
Romar et al. [41]	Case study/2017/Finland.	*N* = 21 (12 from 1st to 3rd grade and 9 from 4th to 6th grade). *Average age (years):* 6–12.	A. Actigraph GT3X and wGT3X+. From waking time to bedtime (7 consecutive days). Evenson et al. (2008), 101 ≤ LPA ≤ 2295; 2296 ≤ AFM ≤ 4011; VPA ≥ 4012.	Traditional school schedule: 4 h 15 min in 1st–3rd grade and 6 h 15 min in 4th–6th grade. During Fridays and outdoor days, the school Schedule: 4 h 15 min. Traditional day include PE sessions.	Traditional day student’s LPA, MVPA and walking distance: 19.6 min/h, 6.9 min/h and 4513 steps. Outdoor day student’s LPA, MVPA and walking distance: 24.3 min/h, 8.8 min/h and 5613 steps.
Rush et al. [42]	Cross-sectional/2009/New Zealand.	*N* = 47 (59.6% girls). *Average age (years):* 8–11.	A. Actical^TM^ and pedometer Digiwalker SW200. During school hours (3 consecutive days). Puyau et al. (2014), LPA ≥ 100 cpm; AFM ≥ 1500 cpm; VPA ≥ 6500.	School schedule: 5 h. No reported PE sessions.	Average students’ LPA: 92.5 min/d (Boys: 89.2 min/d, Girls: 94.8 min/d). Average students’ MVPA: 67.8 min/d (Boys: 68.5 min/d, Girls: 67.4 min/d) Average students walking distance at school: 7424 steps (Boys: 8103 steps, Girls: 6963 steps).
Taylor et al. [43]	Cross-sectional/2016/Skelmesdale (UK).	*N* = 215 (48.8% girls). *Average age (years):* 10.2 (9–10).	A. Actigraph GT9X. During all day (7 consecutive days). 142 ≤ AFM ≤ 464 mg→ 3 ≤ AFM ≤ 5.99 METs; VPA ≥ 6 METs.	School schedule: 6 h 30 min. 15.7 min-recess + 37.9 min lunchtime (Only recess). PE session: 90.7 min.	Boys’ LPA and MVPA: 157.5 min and 20.9 min/d Girls’ LPA and MVPA: 151.9 min and 14.3 min/d.
van Stralen et al. [8]	Cross-sectional/2010/Belgium, Greece, Hungary, Netherland and Switzerland.	*N* = 1025 (51% girls). *Average age (years):* 11.6 (10–12).	A. Actigraph, actitrainers, GT3X and GT1M. From waking time to bedtime (≥ 6 consecutive days). MVPA ≥ 3000 cpm	School schedule: 5 h 54 min. No reported PE sessions.	Average students’ MVPA: 16 min/d.
Watson et al. [44]	Experimental (Pilot cluster randomized controlled trial)/2017/Melbourne (Australia).	*N* = 341 (50% girls in intervention group and 54% girls in control group). *Average age (years):* 9.2 (intervention group) and 9 (control group).	A. Actigraph GT3-X. From waking time to bedtime (7 consecutive days). Freedson et al. (2005), 150 ≤ LPA ≤ 499 cpm; 500 ≤ AFM ≤ 3999 cpm; VPA ≥ 4000 cpm.	School schedule: 6 h 30 min. No reported PE sessions.	At baseline, control group’s MVPA: 34.4 min/d Intervention group: 35.4 min/d.
Zimmo et al. [45]	Cross-sectional/2014/Qatar.	*N* = 92 (57.6% girls). *Average age (years):* 8.8 (boys) and 9.1 (girls).	A. Actigraph wGT3X-BT. During all day (5 consecutive days). Chandler et al. (2015), 101 ≤ LPA ≥ 2295 cpm; MVPA ≥ 2296 cpm; VPA ≥ 4012 cpm.	School schedule: 6 h. 25 min-recess + another 15 min-recess. PE: 2 d/week (45 min).	Average students’ MVPA: 31.8 min/d (Boys: 42.7 min/d, Girls: 23.7 min/d).

A: Accelerometer; cpm: count per minute; d/week: days per week; LPA: Light physical activity; min: minutes; min/d: minutes per day; min/week: minutes per week; MVPA: Moderate to vigorous physical activity; PA: Physical activity; PE: Physical education; VPA: Vigorous physical activity; <: less than someone.

**Table 4:** Characteristics of the analyzed studies with participants of secondary school.

First Author	Study Design/Measure Age/Country	Sample/Age	PA Measures	School Schedule	Results
Aibar et al. [21]	Cross-sectional/2010-12/Huesca (Spain) and Tarbes (France).	*N* = 829 (55.2% girls). *Average age (years):* 14.3.	A. Actigraph GT3X. From waking time to bedtime (8 consecutive days). Evenson et al. (2008), 101 ≤ LPA ≥ 2295 cpm; MVPA ≥ 2296 cpm; VPA ≥ 4012.MVPA ≥ 2296 cpm.	French school schedule: 9 h Spanish: 6 or 8 h. No reported PE sessions.	Average teenagers’ MVPA: Spanish: 23 min/d. French: 28.5 min/d.
Bürgi et al. [23]	Cross-sectional/2013/Winterthur (Switzerland).	*N* = 119 (57.1% girls). *Average age (years):* 12.5 (11–14).	A. Actigraph GT3 X and GPS receiver (BT-Q1000 XT) From waking time to bedtime (7 consecutive days). Evenson et al. (2008), 101 ≤ LPA ≤ 2295 cpm; 2296 ≤ MVPA ≤ 4011 cpm; VPA ≥ 4012.	School schedule: 6 h 30 min. No reported PE sessions.	Average teenagers’ MVPA: 74.7 min/week (Boys: 80.3 min/week, Girls: 71.9 min/week).
Dalene et al. [26]	Cross-sectional/2011/Norway.	*N* = 784 (50.1% girls). *Average age (years):* Group 15 years old = 15.1.	A. Actigraph CT1M and GT3X. From waking time to bedtime (7 consecutive days). Andersen et al. (2006), 100 ≤ LPA ≤ 1999 cpm; MVPA ≥ 2000 cpm.	School schedule: 5 h. No reported PE sessions.	Average teenagers’ PA, LPA and MVPA: Boys: 391 cpm, 69 min/d and 26 min/d. Girls: 393 cpm, 56 min/d and 20 min/d. Girls’ PA < boys.
Gidlow et al. [32]	Cross-sectional/2006-07/England (UK).	*N* = 213 (52.1% girls). *Average age (years):* 14.1 (11–16.5).	A. Actigraph GT1M. From waking time to bedtime (7 consecutive days). Trost et al. (2002) and Puyau et al. (2002), MVPA ≥ 3200 cpm.	School schedule: 6 h. No reported PE sessions.	Average teenagers’ PA and MVPA during school hours: 321.6 cpm and 16.5 min/d.
Grao-Cruces et al. [33]	Longitudinal/2011-12 y 2013-14/Madrid (Spain).	*N* = 658 (49.8% girls). **First measurement.** *Average age:* 13.8. **Second measurement.** *Average age (years):* 15.8.	A. Actigraph CT1M and GT3X. From waking time to bedtime (7 consecutive days). 100 < LPA < 2000 cpm; MVPA > 2000 cpm; VPA > 4000 cpm.	School schedule: 5 h 45 min. 30 min-recess. PE: 2 d/week (45–120 min).	*First measurement:* Boys’ LPA and MVPA: 61.9 min/d and 21.5 min/d. Girls’ LPA and MVPA: 51.8 min/d and 13 min/d. *Second measure:* Boys’ LPA and MVPA: 48.8 min/d and 19.1 min/d. Girls’ LPA and MVPA: 41.4 min/d and 13.3 min/d.
Grao-Cruces et al. [9]	Cross-sectional/2011-12/Madrid (Spain).	*N* = 906 (49.1% girls) *Average age (years):* 14.	A. Actigraph CT1M, GT3X and GT3X+. From waking time to bedtime (7 consecutive days). 100 < LPA < 2000 cpm; MVPA > 2000 cpm; VPA > 4000 cpm.	School schedule: 5 h 45 min. A recess × 30 min. PE: 2 d/week (45–120 min (usually 55 min)) or 1 d/week (90–150 min).	Boys’ PA and MVPA: 83.4 min/d and 22 min/d. Girls’ PA and MVPA: 63.9 min/d and 13.2 min/d.

A: Accelerometer; cpm: count per minute; d/week: days per week; LPA: Light physical activity; min: minutes; min/d: minutes per day; min/week: minutes per week; MVPA: Moderate to vigorous physical activity; PA: Physical activity; PE: Physical education; VPA: Vigorous physical activity; <: less than someone.
